# L-methionine protects against nephrotoxicity induced by methotrexate through modulation of redox status and inflammation

**DOI:** 10.1080/13510002.2023.2270886

**Published:** 2023-11-06

**Authors:** Wessam M. Abdel-Wahab, Nada S. Daifalla, Amina E. Essawy

**Affiliations:** a Department of Zoology, Faculty of Science, Alexandria University, Alexandria, Egypt; b Department of Basic Sciences, Deanship of Preparatory Year and Supporting Studies, Imam Abdulrahman Bin Faisal University, Dammam, Saudi Arabia

**Keywords:** Methotrexate, renal toxicity, redox status, inflammation, hematological parameters, L-methionine, anitoxidant, rats

## Abstract

**Objective:** Methotrexate (MTX) is a drug used in the treatment of cancer and autoimmune disorders; however, its clinical use is limited because of serious side effects including renal toxicity. This study aimed to investigate the protective effect of Lmethionine (L-Met) on MTX toxicity in the kidneys of rats.

**Methods:** Thirty male rats were divided equally into five groups: control (saline), Met400 (400 mg/kg L-Met), MTX (20 mg/kg MTX), MTX-Met300 (300 mg/kg L-Met and 20 mg/kg MTX), and MTX-Met400 (400 mg/kg L-Met and 20 mg/kg MTX). Rats were euthanized one day after the last dose administration (day 16) and serum and renal tissue samples were collected. Renal function and injury indices, oxidative stress/antioxidant indices and proinflammatory cytokines were evaluated.

**Results:** The results showed that L-Met could effectively counteract the nephrotoxic effects of MTX, in a dose-related manner, by improving most of the tested parameters. Furthermore, the higher dose of L-Met was able to restore several parameters to normal levels. In addition, investigation of MTX-induced hematological changes revealed a corrective potential of L-Met.

**Conclusion:** L-Met can be an effective adjuvant therapy to modulate renal toxicity associated with MTX because of its antioxidant and antiinflammatory effects.

## Introduction

Methotrexate (4-amino-10-methylfolic acid; MTX) is a cytotoxic drug structurally related to folic acid and it induces folate deficiency. It has inhibitory effects on the enzyme dihydrofolate reductase, which is required in the synthesis of purine and pyrimidine nucleotides, thus interfering with DNA replication and cell proliferation [1]. In addition, MTX can exert an anti-inflammatory effect by accumulating adenosine and suppressing polyamines [2]. MTX is also known to cause impaired redox system function by generating free radicals, inducing oxidative stress, and being an apoptotic agent targeting T lymphocytes [3]. Due to its opposing effects on proliferation and inflammation, MTX is widely used as an anticancer [4,5] and an anti-inflammatory [6] therapy. MTX is used in high doses (≥500 mg/m^2^) in the treatment of cancer whereas it is administered in low doses in the treatment of nonmalignant and autoimmune pathologies [7,8]. Unfortunately, the cytotoxic effect of MTX can extend to affect other non-targeted malignant tissues causing damage. Because more than 90% of MTX is cleared by the kidney, nephrotoxicity is considered a major complication associated with the use of MTX [9]. MTX and its metabolites can crystallize and precipitate directly in the kidney tubules, decreasing the rate of urine flow [10]. Previous literature reported oxidative stress as a driving force in the nephrotoxicity of MTX through overproduction of free radicals and suppression of the antioxidant defenses [11]. Furthermore, several studies reported inflammation to be a preliminary mechanism in the nephrotoxicity of MTX [9,12]. Since it is becoming increasingly important to enhance the clinical efficacy of therapeutic drugs, an additional therapy is needed to prevent, or at least decrease the toxic effects of MTX drug. Antioxidant supplementation could be beneficial in this regard.

Methionine (Met), a sulfur-containing amino acid, is essential for numerous physiologic functions including metabolism, immune function, and proliferation and differentiation of cells [13]. It is required for the formation of *S*-adenosylmethionine, the main methyl donor in methylation reactions that may affect different cellular macromolecules [14]. Furthermore, Met can affect the oxidative/antioxidant status by acting as a direct and indirect antioxidant [15]. The Met residue of proteins readily reacts with oxygen free radicals and undergoes oxidation to form methionine sulfoxide in a reversible reaction [16]. The cyclic interconversion of Met between oxidized and reduced forms represents a major natural scavenging mechanism against oxidants [17]. Additionally, as a sulfur-containing amino acid, Met acts as the precursor for cysteine, an amino acid essential for the synthesis of the powerful antioxidant glutathione [18]. Several studies reported the anti-inflammatory effect of Met [19,20]. On the basis of the aforementioned beneficial effects, Met may be an appropriate adjunct therapy to be used with MTX. Here, we examine the nephrotoxicity of MTX in rats by assessing kidney function and injury, oxidant, antioxidant, hematological, and inflammatory parameters. Protection against MTX nephrotoxicity by the use of Met has not been reported as of yet. Therefore, our focus in this study is to evaluate the protective efficacy of Met, particularly the L-Met against the nephrotoxicity of MTX in rats.

## Materials and methods

### Chemicals and reagents

Methotrexate and L-methionine (purity >98%) were from Sigma Co. (St Louis, MO, USA). Other kits and reagents were from Bio-Diagnostic Co. (Giza, Egypt).

### Animals and experimental design

Adult male rats (190–220 g) were procured from the animal house facility of Medical Research Institute, University of Alexandria, Egypt. The animals were kept in laboratory conditions (25°C and 12:12-h light/dark cycle) with unrestricted access to the standard rat pellet and tap water throughout the whole experiment duration. They were acclimatized to the laboratory environment for 10 days before starting the experimental protocol. The study design and all methods of animal use were approved by the Alexandria University Animal Ethical Committee (approval number AU04221126301). After acclimatization, groups of 6 rats in each were assigned as follows: Control group: received 0.5 mL distilled water orally by gastric tube once daily for 15 days and 0.5 mL saline intraperitoneally (ip) on day 5 of the treatment protocol, MTX group: received a single ip dose of MTX (20 mg/kg) on day 5 of the experiment [21], L-Met group: treated with 400 mg/kg L-Met orally for 15 days, MTX-Met300 group: treated with L-Met 300 mg/kg orally for 15 days plus a single dose of MTX ip on day 5, and MTX-Met400 group: treated with 400 mg/kg L-Met orally for 15 days plus a single dose of MTX ip on day 5. For the last two groups, L-Met was given one time every day for 15 days starting 4 days before MTX, together with MTX on day 5, and for 10 days after MTX administration. Met dose selection is based on Lin et al. [22].

### Blood sampling and processing of kidney homogenate

Twenty-four hours following the final dose administration (day 16) and after fasting overnight, rats were anesthetized using light diethyl ether prior to euthanization. Blood was collected by decapitation. A portion of the whole blood was obtained into ethylene diamine tetra acetic acid (EDTA) for assessment of hematological parameters using an automated analyzer (Beckman Coulter, USA). The other portion of the blood (without EDTA) was centrifuged at 4000×*g* for 10 min for serum isolation. Serum samples were aliquoted and stored at −80°C to be used in assessing biochemical parameters related to kidney function and injury including blood urea nitrogen (BUN), creatinine (Cr), uric acid (UA), total protein, albumin (Alb), and kidney injury molecule -1 (KIM-1). Kidneys were dissected out, protected from light, and kept in liquid nitrogen until used. For tissue homogenization, kidney samples were removed from the liquid nitrogen, washed in ice-cooled isotonic saline, weighed, and used immediately. One *g* of the kidney tissue was homogenized in ice-cooled phosphate buffered saline (10% w/v) and centrifuged for 10 min at 4000×*g* at 4°C. Aliquots of the collected supernatants were stored at −80°C and were used for the measurement of oxidative stress and inflammatory parameters.

### Calculation of body weight change and relative weight of kidney

Change in body weight (%) = [final body weight (g)-initial body weight (g)/initial body weight (g)] × 100. Relative kidney weight = [weight of the kidney (g)/body weight (g)]× 100.

### Determination of kidney function and injury indices

Levels of BUN, Cr, UA, total protein, and Alb were calorimetrically measured in serum samples using commercial kits (Bio-Diagnostic, Egypt) as described in the manufacturer’s protocols. KIM-1 was measured also in serum samples using ELISA kit.

### Assessment of redox status biomarkers

Renal reactive oxygen species (ROS) level was determined using ELISA kit as described in the manufacturer’s protocols. Renal malondialdehyde (MDA) as a marker for lipid peroxidation, protein carbonyl (PC) as an indicator for protein oxidation, and nitric oxide (NO) were measured according to Ohkawa et al. [23], Levine et al. [24], and Green and Vande Zande [25], respectively. Reduced glutathione (GSH) and oxidized glutathione (GSSG) were measured as described by Beutler et al. (26) and Anderson (27), GSH/GSSG ratio was determined as an indicator of the redox potential. The activity of GPx [28], SOD [29], and CAT [30] were determined in renal tissue.

### Estimation of inflammatory mediators

Quantification of interleukin-1 beta (IL-1β), interleukin-6 (IL-6), and tumor necrosis factor alpha (TNF-α) in kidney homogenates was performed using enzyme-linked immunosorbent assay (ELISA) kits following the manufacturer’s instructions.

### Statistical analysis

The statistical package of social science (SPSS, version 28) was used for statistical analysis. Data are presented as mean ± standard error of the mean (SEM). After data normalization, statistical comparisons of different experimental groups were performed by one-way analysis of variance (ANOVA) followed by Tukey’s post hoc test for pairwise comparisons for all groups. For the analysis of body weight, TNF-α, and GSH/GSSG ratio, Kruskal-Wallis non-parametric test was applied as the data failed to be normalized. A *P* value of <0.05 was considered statistically significant.

## Results

### Effect of MTX with and without L-Met on body weight and relative kidney weight of rats

**Figure 1.**
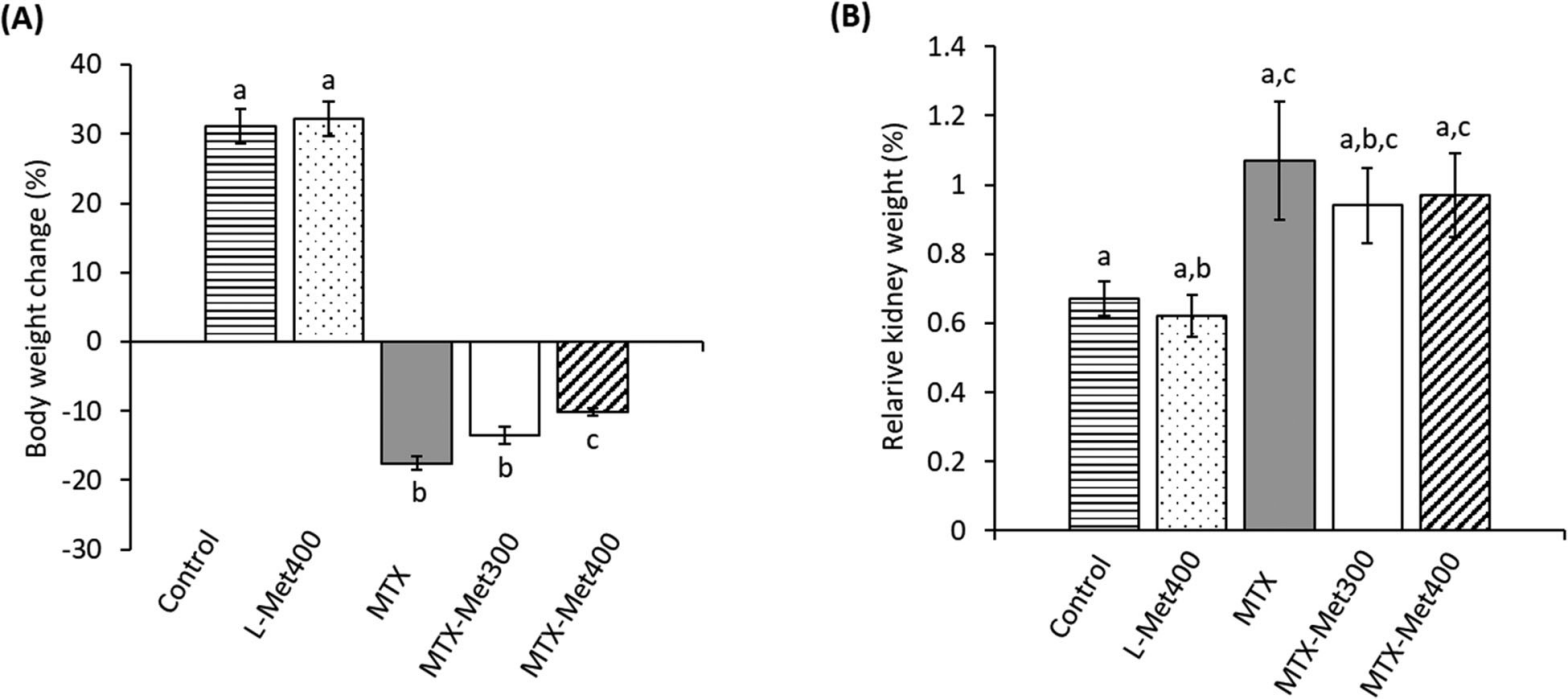
Changes in body weight (A) and kidney weight (B) of rats treated with MTX with or without L-Met. Values are presented as mean ± SEM (*n* = 6). Significant differences (*P *< 0.05) between groups were indicated by different letters above the error bars. Similar letters above the error bars indicate no significant differences. Abbreviations: L-Met, L-methionine; MTX, methotrexate.

The changes in body and relative kidney weights are depicted in Figure 1. Rats treated with MTX exhibited a significant reduction of 43.5% in body weight (*p* < 0.001) but an insignificant increase of 59.7% in the relative kidney weight in comparison with the control group. Treatment of rats receiving MTX with 300 mg/kg L-Met improved body weight, although the value did not show a significant difference from the MTX group. On the other hand, the higher dose of L-Met (400 mg/kg) significantly improved the body weight gain compared to the MTX group (*p* < 0.05). No significant changes in relative kidney weight were observed after treatment of the MTX-injected group with any of the two doses of L-Met compared to the MTX-only group.

### Effect of MTX with and without L-Met on kidney dysfunction and injury

Table 1 illustrates the effect of MTX on kidney function and injury parameters in different groups. The data showed that MTX significantly (*p* < 0.001) raised the levels of BUN, Cr, UA, and KIM-1 by 151.4%, 341%, 223%, and 201.7% respectively, while it significantly (*p* < 0.05) lowered Alb and protein levels by 46.2% and 30.7% below the control group, respectively. Administration of L-Met at the two selected doses significantly improved these parameters. At the lower dose (300 mg/kg), L-Met decreased BUN, Cr, UA, and KIM-1 by 33.4%, 22.1%, 31.1%, and 25.1% respectively, as compared with MTX group. Meanwhile, the higher dose of L-Met (400 mg/kg) showed a reduction of 40%, 45.5%, 51.6%, and 50.8% respectively, for BUN, Cr, UA, and KIM-1 levels compared to the MTX group. The two doses of L-Met restored the Alb level to normal although its level was not significantly different from the MTX alone group. In addition, both doses of L-Met improved the total protein level compared with the MTX group, although the difference was not significant between any of the experimental groups.

**Table 1.** Changes in serum biochemical parameters related to kidney function and injury under the influence of MTX with and without L-Met.

Group	KIM-1 (pg/ml)	BUN (mg/dl)	Cr (mg/dl)	UA (mg/dl)	Protein (mg/dl)	Alb (mg/dl)
Control	108.300^a^ ± 10.99	27.583^a^ ± 1.20	0.480^a^ ± 0.05	3.483^a^ ± 0.34	7.600^a^ ± 0.68	4.550^a^ ± 0.52
L-Met400	104.267^a^ ± 6.19	27.100^a^ ± 2.06	0.447^a^ ± 0.04	3.067^a^ ± 0.35	7.417^a,b^ ± 0.77	4.750^a ^± 0.43
MTX	326.783^b^ ± 16.88	69.350^b^ ± 3.40	2.117^b^ ± 0.36	11.250^b^ ± 0.71	5.267^b^ ± 0.53	2.450^b^ ± 0.44
MTX-Met300	244.725^c^ ± 17.48	46.200^c^ ± 1.86	1.650^b,c^ ± 0.22	7.750^c^ ± 0.64	6.233^a,b^ ± 0.53	2.850^b,c^ ± 0.25
MTX-Met400	160.867^d^ ± 10.12	41.717^c^ ± 2.67	1.152^c^ ± 0.18	5.450^a,c^ ± 0.37	6.617^a,b^ ± 0.51	3.650^a,b^ ± 0.41
One Way ANOVA F (*p*)	52.04 (<0.001)	33.258 (<0.001)	24.595 (<0.001)	37.93 (<0.001)	3.083 (<0.05)	7.723 (<0.001)

Notes: Values are presented as mean ± SEM (*n* = 6). The mean values within the same column that have different superscripts (a–d) are significantly different (*P *< 0.05). Abbreviations: Alb, albumin; BUN, blood urea nitrogen; Cr, creatinine; KIM-1, kidney injury molecule -1; L-Met, L-methionine; MTX, methotrexate; UA, uric acid.

### Effect of L-Met on MTX-induced changes in renal oxidative status

**Figure 2.**
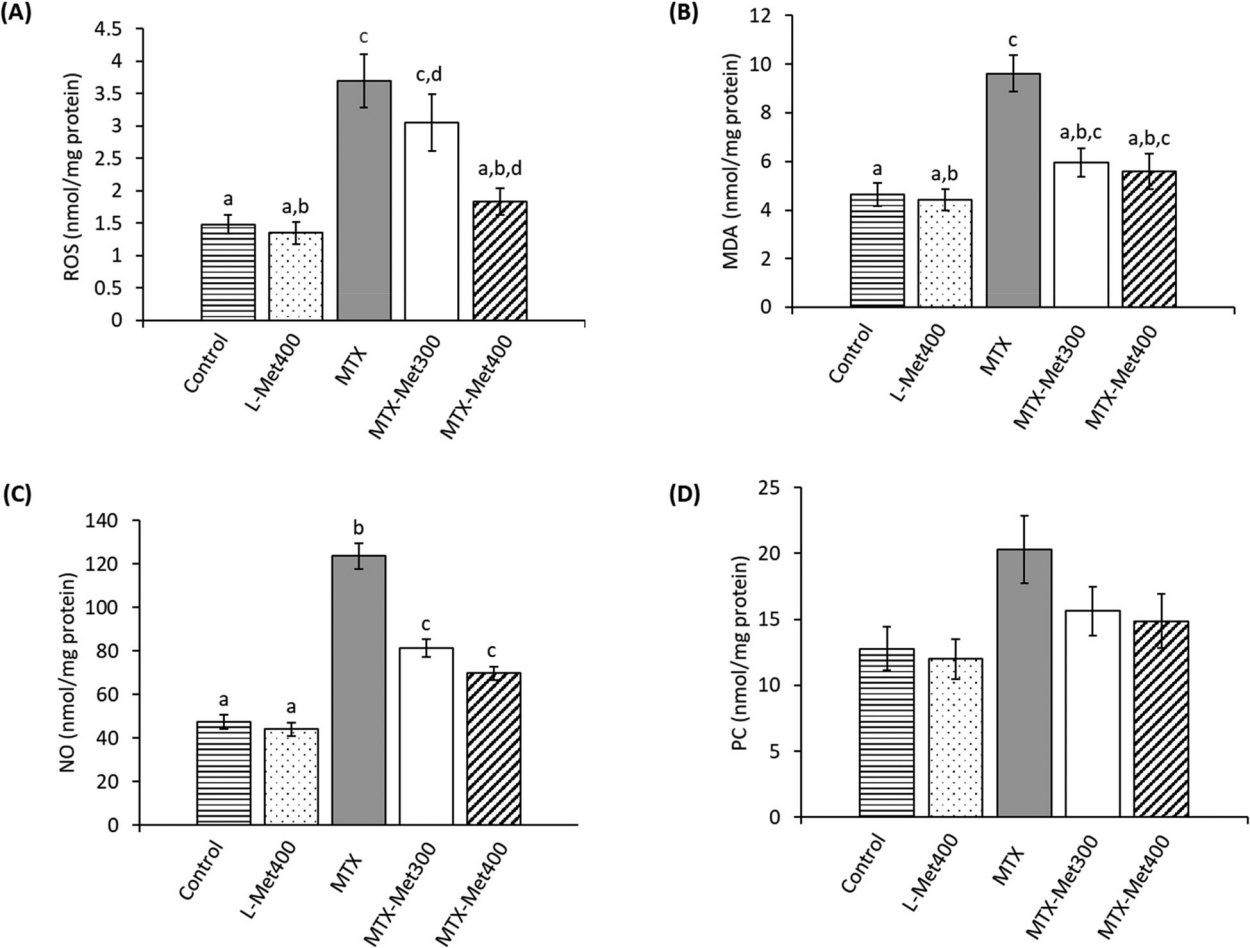
Oxidative markers in rats treated with MTX with and without L-Met. Renal ROS (A); MDA (B); NO (C) and PC (D) levels were evaluated for different groups. Values are presented as mean ± SEM (*n* = 6). Significant differences (*P *< 0.05) between groups were indicated by different letters above the error bars. Similar letters above the error bars indicate no significant differences. Abbreviations: L-Met, L-methionine; MTX, methotrexate; MDA, malondialdehyde; NO, nitric oxide; PC, protein carbonyl; ROS, reactive oxygen species.

To evaluate the state of oxidation in the different groups, renal levels of ROS, MDA, NO, and PC were assessed (Figure 2). Administration of MTX significantly increased the renal level of ROS by 149.5% (*p *< 0.001), MDA by 106.8% (*p* < 0.01), and NO by 160.4% (*p* < 0.001) relative to the control group. L-Met treatment significantly and dose-dependently reduced ROS, MDA, and NO by 17.6%, 38%, and 34.2% for the lower dose of L-Met and by 50.5%, 42%, and 43.5 for the higher dose compared to the MTX group. Compared to the control group, the rats receiving MTX showed an increase of 58.7% in PC which was decreased by 23% and 26.7% by the lower and higher dose of L-Met, respectively. However, the change in PC level was not significant. Notably, the administration of L-Met (400 mg/kg) alone had no effect on the levels of nearly all tested oxidative parameters.

### L-Met ameliorates MTX-induced alteration in renal antioxidant defenses in rats

**Figure 3.**
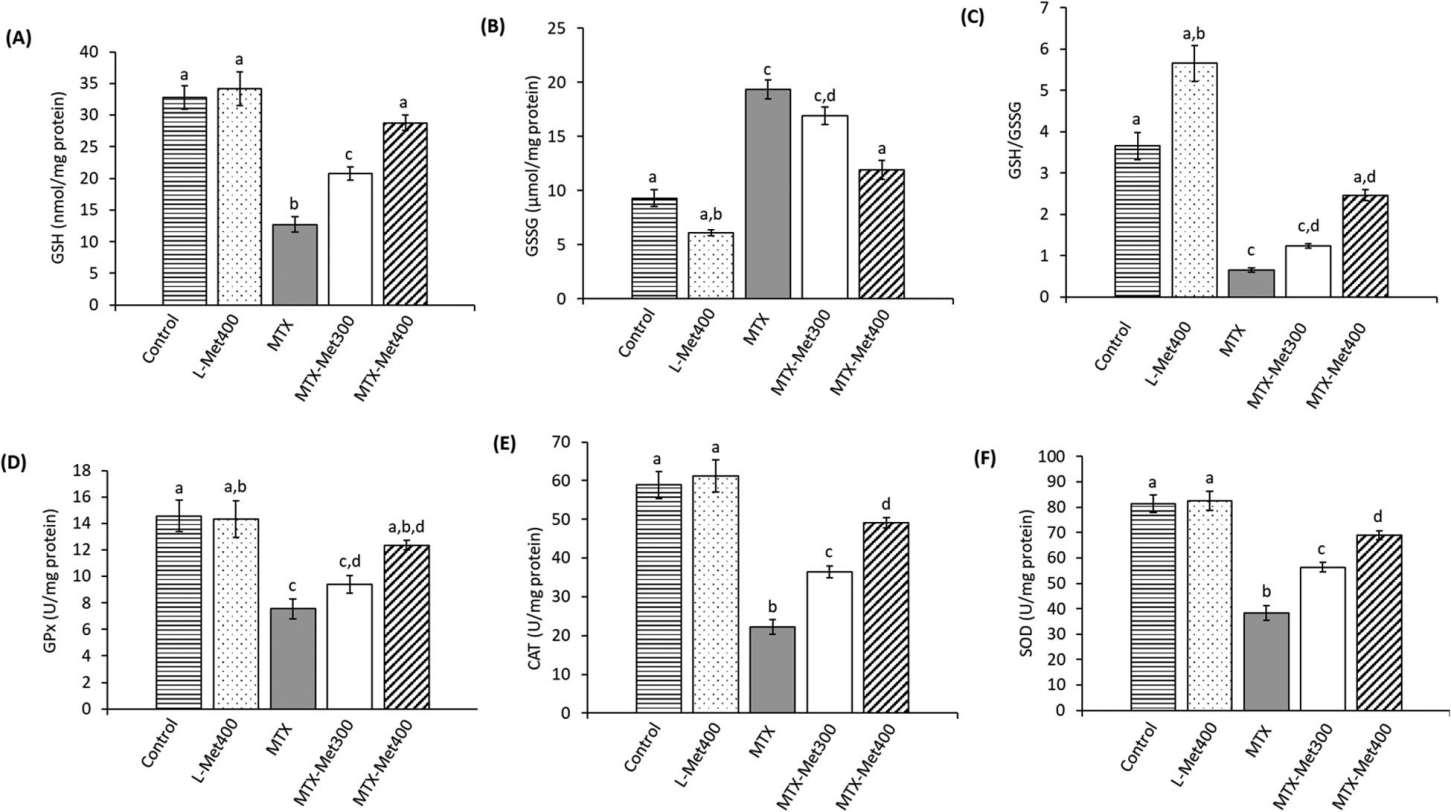
Antioxidant markers in rats treated with MTX with and without L-Met. Different groups were evaluated for their level of renal GSH (A); GSSG (B), GSH/GSSG ratio (C), the activities of GPx (D); CAT (E); and SOD (F). Values are presented as mean ± SEM (*n* = 6). Significant differences (*P *< 0.05) between groups were indicated by different letters above the error bars. Similar letters above the error bars indicate no significant differences. Abbreviations: CAT, catalase; GPx, glutathione peroxidase; GSH, reduced glutathione; GSSG, oxidized glutathione; L-Met, L-methionine; MTX, methotrexate; SOD, superoxide dismutase.

The results showing the antioxidant status of the kidney under the influence of MTX with or without L-Met are shown in Figure 3. The MTX group showed a significant decrease of 61.2% in the renal GSH content compared to the control group (*p* < 0.001) which was associated with a significant increase of 108.62% in GSSG content (*p* < 0.001). The GSH/GSSG ratio which indicates the glutathione redox state also decreased significantly (by 82.11%) in the MTX group compared to the control group (*p* < 0.001). Furthermore, a reduction of 48.2%, 52.9%, and 62.2% in the activity of GPx, SOD, and CAT, respectively, relative to the control group (*p* < 0.001) was detected. Both doses of L-Met markedly increased antioxidant defenses in the kidney of rats administered with MTX toward its normal value. In particular, the higher dose of L-Met (400 mg/kg) was more effective than the lower dose in restoring the antioxidant status indices compared to the MTX group. In L-Met alone treated group, the measured antioxidants remained almost the same as the normal control.

### L-Met ameliorates the changes in pro-inflammatory cytokines induced by MTX

**Figure 4.**
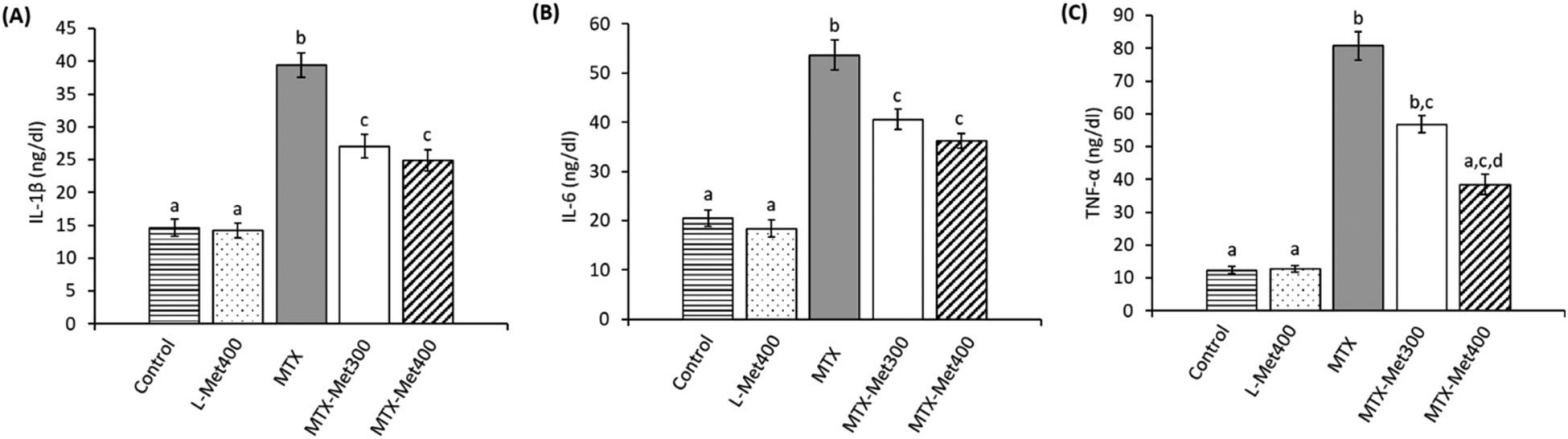
Inhibitory effect of L-Met on MTX-induced inflammatory cytokines in rats. Different groups were assessed for their levels of IL-1β (A); IL-6 (B); and TNF-α (C). Values are presented as mean ± SEM (*n* = 6). Significant differences (*P *< 0.05) between groups were indicated by different letters above the error bars. Similar letters above the error bars indicate no significant differences. Abbreviations: IL, interleukin; L-Met, L-methionine; MTX, methotrexate; TNF, tumor necrosis factor.

According to Figure 4, MTX elicited a significant (*p* < 0.001) increase of 170%, 161.7%, and 552.9% in levels of IL-1β, IL-6, and TNF-α, respectively, compared to the control group. Treatment of rats with L-Met showed an anti-inflammatory effect, as indicated by decrease in the measured cytokines compared to the MTX-only group. Treatment of rats administered MTX with 300 mg/kg L-Met induced significant decrease of 31.4%, and 24.4% in IL-1β, and IL-6 levels, respectively, compared to the MTX group (*p* < 0.001). Receiving the lower dose of L-Met caused a 29.7% decrease in TNF-α as compared to the MTX group, however, the difference was not significant. Furthermore, treatment with the highest dose (400 mg/kg) caused significant decrease of 36.9%, 32.4%, and 52.4%, in IL-1β (*p* < 0.001), IL-6 (*p* < 0.001), and TNF-α (*p* < 0.01) levels respectively, compared to the MTX group.

### Effect of L-Met on MTX-induced changes in hematological parameters

To assess any suppressive effect of MTX on the numbers of rats’ blood cells and to investigate the corrective capability of L-Met, the count of red blood cells (RBCs), white blood cells (WBCs), and platelets was determined in different experimental groups (Table 2). The data showed a decrease in the number of RBCs and WBCs in rats injected with MTX compared to the control group; however, the decrease in cell numbers was not statistically significant. Only platelet count decreased significantly compared to the control group (*p* < 0.01). Both doses of L-Met restored the platelet count to a number comparable to the control group. We opt to perform the differential leucocytic count (Table 3) in the different groups to examine if there is any obvious link between a specific subtype of leukocytes and the change in WBCs counts and the toxic effects of MTX. Our results illustrated that MTX decreased the percentages of lymphocytes, monocytes, basophils, and eosinophils compared to controls. However, the difference was not significant except for basophil numbers (*p* < 0.05). On the other hand, MTX significantly increased the neutrophils percentages, as compared to controls (*p* < 0.001). Both L-Met doses restored the lymphocyte count to normal, while the restoration of the neutrophil count to normal was achieved only with the higher dose.

**Table 2.** Changes in rats’ blood cell counts under the influence of MTX with and without L-Met.

Group	RBCs	WBCs	Platelets
Control	7.350^a^ ± 0.59	9.367 ± 0.75	480.550^a^ ± 30.55
L-Met400	7.867^a,b^ ± 0.50	10.017 ± 0.69	495.483^a,b^ ± 33.42
MTX	6.167^a,c^ ± 0.28	8.133 ± 0.44	305.517^c^ ± 33.42
MTX-Met300	6.200^a,b,c^ ± 0.29	8.533 ± 0.70	338.750^a,c^ ± 30.39
MTX-Met400	7.050^a,b,c^ ± 0.39	9.067 ± 0.76	408.767^a,b,c^ ± 32.15
One Way ANOVA F (*p*)	3.141 (<0.05)	1.502 (0.233)	6.462 (<0.001)

Notes: Values are presented as mean ± SEM (*n* = 6). The mean values within the same column that have different superscripts (a, b, c) are significantly different (*P *< 0.05). Abbreviations: L-Met, L-methionine; MTX, methotrexate; RBCs, red blood cells; WBCs, white blood cells.

**Table 3.** Changes in the differential leucocytic count of rats under the influence of MTX with and without L-Met.

Group	Lymphocytes %	Monocytes %	Neutrophils %	Basophils %	Eosinophils %
Control	46.133 ± 1.51	5.267 ± 0.45	40.950^a^ ± 1.13	0.652^a^ ± 0.09	2.120 ± 0.13
L-Met400	45.483 ± 2.54	5.700 ± 0.50	42.000^a^ ± 1.30	0.692^a,b^ ± 0.05	2.250 ± 0.20
MTX	38.050 ± 1.51	4.100 ± 0.49	53.067^b^ ± 2.75	0.400^a,c^ ± 0.06	1.617 ± 0.15
MTX-Met300	39.717 ± 1.25	4.517 ± 0.47	51.583^b^ ± 2.79	0.475^a,b,c^ ± 0.06	1.733 ± 0.19
MTX-Met400	43.317 ± 2.48	5.033 ± 0.52	45.183^a,b^ ± 2.51	0.600^a,b,c^ ± 0.04	1.917 ± 0.20
One Way ANOVA F (*p*)	2.897 (<0.05)	1.723 (0.178)	5.539 (<0.01)	4.135 (<0.05)	2.280 (<0.01)

Notes: Values are presented as mean ± SEM (*n* = 6). The mean values within the same column that have different superscripts (a–c) are significantly different (*P *< 0.05). Abbreviations: L-Met, L-methionine; MTX, methotrexate.

## Discussion

Despite the remarkable therapeutic effects of the MTX drug, its clinical applications are challenged by its wide spectrum of adverse side effects and toxicities including nephrotoxicity and hematological toxicity [31]. Therefore, there is growing interest in finding ways to palliate the damaging effects of MTX while maintaining its therapeutic efficacy. Nephrotoxicity is a serious adverse effect that accompanies the use of MTX as it can significantly augment MTX toxicity. Therefore, it is pivotal to examine the toxic effects of MTX and try to prevent its induced damage. Several reports have identified oxidative stress as one of the primary mechanisms involved in the toxicity of MTX [11,32]. Additionally, MTX treatment has been reported to induce proinflammatory cytokines synthesis [33,34]. The results of the current study confirmed the nephrotoxicity of MTX which was elucidated by elevation in renal function and injury biomarkers, imbalance in redox status, excessive production of inflammatory mediators, and alteration in the hematological parameters. Protection against MTX-induced nephrotoxicity may be an approach to enable the widening of its therapeutic use. Because oxidative stress and inflammation are the underlying mechanisms of MTX-induced nephrotoxicity, the use of agents with antioxidant and anti-inflammatory properties may be a potential approach to boost the clinical efficacy of MTX. Met has been reported to protect against the nephrotoxicity of gentamicin [35], cisplatin [22], and polymyxin [36]. Herein, we examined the nephroprotective effect of L-Met on the toxic effects of MTX. The results presented revealed a promising protective effect of L-Met mainly due to its antioxidant and anti-inflammatory actions.

Evaluation of changes in body and organ weight may indicate toxicity after exposure to drugs. A significant decrease in the body weight of rats administered MTX was observed in the current study which agrees with Patel et al. [37]. In addition, there was a 0.6-fold increase in the relative kidney weight in rats caused by the administration of MTX. Treatment with L-Met significantly mediated body weight gain, probably due to increased food consumption and digestibility. Our results are in line with a previous report on anti-anorexic effect of D-Met against body weight loss and anorexia induced by the anticancer drug cisplatin [22]. Furthermore, the increase in relative kidney weight induced by MTX was slightly less in the presence of L-Met.

Elevation of BUN, Cr, and UA levels clearly indicate impaired elimination of wastes by the kidney. KIM-1, a transmembrane protein significantly upregulated in kidney injury especially injury of renal proximal tubules, is a specific and sensitive marker of nephrotoxicity (38). Therefore, increased levels of the aforementioned biomarkers diagnose renal dysfunction and injury. Several studies reported similar increase in renal function indices and injury after MTX administration [12,39,40]. Consistent with these results, the current study revealed a significant increase in BUN, Cr, UA, and KIM-1 levels with a decrease in the level of peotein and Alb after MTX administration. The increase in KIM-1 may be considered as a compensatory mechanism because it can inhibit apoptosis and participate in re-epithelization of kidney tubules [41]. The poor solubility and precipitation of MTX and its metabolites in kidney tubules causing a decrease in renal clearance may be the reason for the deterioration of kidney function in treated rats [10]. In addition, MTX can have a direct toxic effect on cells of the renal tubules due to its ability to inhibit the dihydrofolic reductase enzyme, an enzyme involved in the activation of folic acid [42]. Loss of kidney function in rats treated with MTX may also be closely associated with increased ROS production, which can attack membrane lipids, leading to damage to tubular cells and glomeruli [43]. Oxidative damage has been reported to be common in several models of nephrotoxicity, which can point to the glomerulus and the proximal tubule as the main targets of radical attack [44]. The reduction in total protein and Alb levels with MTX administration reported herein may be due to decreased synthesis or increased metabolism of plasma proteins [45]. Remarkably, L-Met afforded protection and mitigated renal function in rats treated with MTX, indicating a renoprotective effect. It could effectively decrease BUN, Cr, UA, and KIM-1 levels.

Due to their high metabolic activity and energy need to support active transport and reabsorption of substances, renal tubules are more susceptible to the formation and attack of free radicals [46]. Perturbation in renal redox homeostasis is a frequent adverse effect of MTX administration. The results reported here strongly support oxidative stress as a preliminary mechanism in MTX nephrotoxicity. A marked elevation in oxidative status was observed after MTX administration, as indicated by increased renal ROS, MDA (an indicator of lipid peroxidation) and PC (a reliable marker of protein oxidation). MTX has been reported to negatively affect the mitochondrial machinery and enhances the generation of ROS [11] which can in turn stimulate oxidative damage of important subcellular macromolecules including lipids and proteins. This may explain the observed increase in the level of oxidation to lipids and proteins. Additionally, the level of NO was increased in our study which corroborates previous studies [9,40,47]. NO is a powerful free radical that can react and damage lipids, proteins, and DNA when it is produced in excess. Furthermore, NO can amplify the effects of ROS by reacting with superoxide radicals and exaggerates the generation of peroxynitrite, a strong oxidizing agent that can further contribute to the damage of lipids and proteins [48]. Craven et al. [49] reported that oxidative stress can induce the release of a variety of vasoactive mediators that cause renal vasoconstriction and reduce the rate of glomerular filtration. This may explain the aforementioned MTX-induced impairment in renal function. L-Met administration to rats treated with MTX significantly abrogated the production of oxygen species and the oxidation of lipid and protein as indicated by lower levels of ROS, MDA, PC, and NO. The protective effect of L-Met may be closely associated with its potential as a radical scavenger. L-Met has been reported to scavenge ROS/RNS and detoxify oxidants by conveying electrons and hydrogen and the formation of radical adducts [50]. Due to its chemical structure as sulfur-containing amino acid, ROS can easily oxidize L-Met to methionine sulfoxide (Met-O); however, this reaction is reversible and Met-O can be converted back to Met in a reaction catalyzed by the methionine sulfoxide reductases (MSRs) family [16]. This mechanism enables L-Met to work as a scavenger that consumes ROS in each oxidation–reduction cycle. Furthermore, L-Met plays a vital role in enriching cellular antioxidant defenses [51].

The disposal of free radicals requires an effective system of antioxidants, either nonenzymatic (such as GSH) or enzymatic (including GPx, SOD, and CAT). Depletion of any of these components due to increased oxidative stress can lead to pathophysiological changes. Glutathione is a major endogenous antioxidant against oxidative stress that is present in the reduced (GSH) and oxidized (GSSG) forms. An alteration in the redox state due to exposure to ROS can imbalance the GSH/GSSG ratio. In our study, the marked decrease in GSH/GSSG ratio in the kidney of rats treated with MTX indicates the occurrence of oxidative stress. The observed decrease in GSH/GSSG ratio results from the decrease in GSH level with a corresponding increase in GSSG level. Furthermore, the activity of GPx, SOD, and CAT was decreased in rats treated with MTX. These results further support the role of oxidative stress in MTX nephrotoxicity. Similar deterioration in the antioxidant system has previously been reported [12,40,47]. Overconsumption of antioxidant components during elimination of the upregulated radical formation may explain the suppression of the antioxidants after MTX administration. NO is a potent radical that can exaggerate the production of a variety of free radicals which in turn can contribute to the consumption of the antioxidants [52]. NO can inhibit the activity of catalase and cause the accumulation of hydrogen peroxide which consequently reacts with superoxide radical to generate the more robust hydroxyl radicals [9,53]. Increased NO can also affect cellular GSH negatively and consequently intensify renal oxidative damage [54]. Inhibition of ROS generation along with the enrichment of endogenous antioxidants can ameliorate MTX nephrotoxicity. Results reported herein showed a marked recovery in the antioxidative status of the kidney upon treatment with L-Met. Met has been reported to be the precursor of homocysteine and cysteine which are essential substrates for the synthesis and regeneration of important antioxidants such as glutathione [18].

Excessive production of pro-inflammatory cytokines has been found to play a pivotal role in the pathogenesis of renal dysfunction [55]. Accordingly, inflammation may be a prominent mechanism in the initiation and progression of MTX nephrotoxicity. The findings of our study strongly support this hypothesis as indicated by a marked elevation in the level of key pro-inflammatory mediators namely, IL-1β, IL-6, and TNF-α in the kidney. These results are consistent with Aladaileh et al. [53], Elsawy et al. [21], and Younis et al. [47]. Nuclear factor-kappa B (NF-κB) is essential for the transcrip­tion of the pro-inflammatory genes and thus induction of inflammation. Enhanced ROS production has been found to activate NF-κB which subsequently promotes up-regulation of genes encoding the synthesis of pro-inflammatory cytokines [56]. Met has been shown to exert anti-inflammatory effects [19,20]. Therefore, the protective effect of L-Met seen in our study could have been attributed, in part, to its inhibitory effect on the production of the tested pro-inflammatory cytokines, which are associated with MTX-induced renal toxic insults.

Previous studies reported a decrease in blood cell numbers following MTX treatment in humans and rats [1,37]. Indeed, our data showed that MTX treatment reduced red blood cell, white blood cell, and platelet counts. The inhibitory effect of MTX on blood cell proliferation might be attributed to its binding to dihydrofolate reductase which interferes with the production of nucleotides and DNA synthesis leading to the suppression of cell survival and proliferation [1]. Paul et al. [57] reported that MTX induces platelet apoptosis via JNK-mediated mitochondrial damage. Our data also revealed a decline in different leukocytes including lymphocytes, monocytes, basophils, and eosinophils. Surprisingly, the number of neutrophils in our study increased with MTX, contrary to previous studies. This discrepancy in the effect of MTX on neutrophils could have been caused by differences in the MTX dose, the route of injection, and the timing of sample collection. It is noteworthy that MTX restored neutrophil numbers in patients suffering from neutropenia, associated with Felty’s syndrome, by lowering the numbers of IgG-reactive neutrophils [58]. Investigation of the number of IgG-reactive neutrophils before and after MTX administration may provide an insight on possible mechanisms of the rise in number of neutrophils seen in our study. The MTX-induced inflammatory environment might have promoted the production of more neutrophils. It was reported that IL-1β and TNF-α can trigger the production of neutrophils [59]. Our findings revealed the corrective effects of L-Met on the number of blood cells. Met-induced increase in cell numbers is most likely due to its suppressive effect on oxidants and the consequent decrease of cell apoptosis. The mechanism by which Met increased the number of neutrophils is not clear. We hypothesize that L-Met corrective effect on neutrophil numbers could be indirect through the inhibition of IL-1β and TNF-α which changes the inflammatory milieu to an environment less favorable for neutrophil proliferation.

In conclusion, our data showed that L-Met can inhibit the deleterious effects of MTX on kidneys of rats by counteracting the damaging effects caused by the increased activities of the oxidative and inflammatory systems. Therefore, L-Met can be considered a promising agent in the management of nephrotoxicity associated with MTX treatment. A limitation of this study is the lack of histopathological examination and some molecular analysis. Therefore, further studies including histopathological examination to confirm the renal injury and some molecular analysis such as gene expression of antioxidant enzymes and apoptosis to further elucidate the nephrotoxicity MTX and have a deep insight on the exact protective mechanism(s) of L-Met are recommended.

## Data Availability

The data used to support the findings of this study are available from the corresponding author upon request.
